# Rapid detection of colistin resistance protein MCR-1 by LC–MS/MS

**DOI:** 10.1186/s12014-019-9228-2

**Published:** 2019-02-26

**Authors:** Honghui Wang, Yong Chen, Jeffrey R. Strich, Steven K. Drake, Jung-Ho Youn, Avi Z. Rosenberg, Marjan Gucek, Patrick T. McGann, Anthony F. Suffredini, John P. Dekker

**Affiliations:** 1 Critical Care Medicine Department, Clinical Center, National Institutes of Health, Bethesda, MD USA; 2 Proteomics Core Facility, National Heart, Lung and Blood Institute, National Institutes of Health, Bethesda, MD USA; 3 Department of Laboratory Medicine, Clinical Center, Microbiology Service, National Institutes of Health, 10 Center Drive, Bethesda, MD USA; 4 Kidney Disease Section, National Institute of Diabetes and Digestive and Kidney Diseases, National Institutes of Health, Bethesda, MD USA; 5 Department of Pathology, Johns Hopkins University, Baltimore, MD USA; 6 Walter Reed Army Institute of Research, Silver Spring, MD USA; 7 Laboratory of Clinical Immunology and Microbiology, National Institute of Allergy and Infectious Diseases, Bethesda, MD USA

## Abstract

**Background:**

Colistin (polymyxin E) and polymixin B are important bactericidal antibiotics used in the treatment of serious infections caused by multi-drug resistant Gram-negative organisms. Transferrable plasmid-mediated colistin resistance, conferred by the product of the *mcr*-*1* gene, has emerged as a global healthcare threat. Consequently, the rapid detection of the MCR-1 protein in clinical bacterial isolates has become increasingly important. We used a genoproteomic approach to identify unique peptides of the MCR-1 protein that could be detected rapidly by liquid chromatography tandem mass spectrometry (LC–MS/MS).

**Methods:**

MCR-1 tryptic peptides that were efficiently ionized and readily detectable were characterized in a set of *mcr*-*1*-containing isolates with triple quadrupole LC–MS. Three optimal peptides were selected for the development of a rapid multiple reaction monitoring LC–MS/MS assay for the MCR-1 protein. To investigate the feasibility of rapid detection of the MCR-1 protein in bacterial isolates using this assay, a blinded 99-sample test set was built that included three additional *mcr*-*1*-containing clinical isolates tested in triplicate (9 samples) and 90 negative control isolates.

**Results:**

All of the *mcr*-*1*-containing isolates in the test set were accurately identified with no false positive detections by three independent, blinded operators, yielding an overall performance of 100% sensitivity and specificity for multiple operators. Among the three peptides tested in this study, the best performing was DTFPQLAK. The isolate-to-result time for the assay as implemented is less than 90 min.

**Conclusions:**

This work demonstrates the feasibility of rapid detection of the MCR-1 protein in bacterial isolates by LC–MS/MS.

**Electronic supplementary material:**

The online version of this article (10.1186/s12014-019-9228-2) contains supplementary material, which is available to authorized users.

## Background

The rise of carbapenem-resistant gram-negative infections has led to an increase in the use of the polymixin B and colistin because of limited effective alternative antibiotic treatment [1]. Transferrable plasmid-mediated colistin resistance, conferred by the product of the *mcr*-*1* gene, was first described in 2015 in China and has since emerged as a global threat [2–7]. Colistin resistance has been associated with increased risk of in-hospital mortality, and outcomes of patients infected with *mcr*-*1*-containing isolates have shown a trend towards increased 30-day mortality [8, 9].

Detection of colistin resistance is thus of increasing clinical importance. Most clinical testing is based on phenotypic methods that are difficult to standardize and may require 18–24 h for completion following sub-culture of a clinical isolate. Rapid (2 h) assays that can be performed on clinical isolates have been developed recently based on the detection of acidic products from bacterial metabolism in the presence of colistin or polymyxin B [10–13]. While these assays are sensitive and specific, they do not provide information on the mechanism of resistance, which may become important if MCR-1 allele-specific inhibitors are introduced to the market [14]. Other phenotypic tests of colistin resistance based on inhibition of MCR-1 activity by EDTA have been developed and may differentiate MCR-1 from other mechanisms [12, 15]. Additionally, real-time PCR assays have been developed for the *mcr*-*1* gene and related variants [16–18].

We have previously employed a genoproteomic approach to select and validate optimal tryptic peptides for the detection of the *Klebsiella pneumoniae* carbapenemase protein (KPC) by LC–MS/MS [19]. In the current study, we used a similar approach to define unique tryptic peptides for MCR-1. Highly sensitive Orbitrap Lumos LC–MS/MS, employing both data dependent analysis (DDA) and targeted approaches, was used to identify efficiently ionized and reliably detected unique MCR-1 tryptic peptides for assay development. A standard multiple reaction monitoring (MRM) assay was developed using the Agilent ChipCube triple quadruple (QQQ) instrument with labelled peptides for the rapid detection of a set of three MCR-1 peptide markers, followed by a feasibility study.

## Methods

*Bacterial isolates* 92 de-identified, sub-cultured clinical bacterial isolates were used as negative control samples divided between assay development and test sets (Table 1).

**Table 1:** *mcr-1*-positive and negative control isolates used in assay development and feasibility testing

Name	Assay development		Feasibility testing	
	Negative control	*mcr-1*-containing	Negative control	*mcr-1*-containing
*Achromobacter* sp.			4	
*Aeromonas* sp.			1	
*Citrobacter freundii complex*			5	
*Citrobacter koseri*			2	
*Chryseobacterium* sp.			1	
*Enterobacter cloacae* complex			9	
*E. coli*		5*	23	1**
*Enterococcus faecalis*			1	
*Klebisella oxytoca*			4	
*K. oxytoca/Raoutella ornitholytica*			1	
*Klebsiella pneumonaie*			12	2**
*Morganella morganii*			1	
*Pseudomonas aeruginosa*	1		12	
*Proteus mirabilis*			1	
*Pantoea* sp.	1		0	
*Rhizobium radiobacter*			2	
*Stenotrophomonas maltophilia*			5	
*Serratia liquifaciens*			1	
*Serratia marcescens*			2	
*Sphingomonas* sp.			1	
*Staphylococcus epidermidis*			1	
*Staphylococcus haemolyticus*			1	
Total	2	5*	90	3**

*4 of the isolates are from the same patient, **in triplicate for each isolate

A subset of these negative control isolates has been previously described in the development of a previously published proteomic assay [19]. Eight previously sequenced *mcr*-*1*-containing isolates were obtained from the WRAIR Multidrug Resistant Organism Repository and Surveillance Network (MRSN) collection [2, 20]. The presence of the intact *mcr*-1 gene in these isolates had been determined previously by whole genome sequencing performed at WRAIR (not part of the present study). The identities of all isolates used in this study were re-confirmed by MALDI-TOF MS (Bruker MicroFlex LT mass spectrometer, Bruker Daltonics, Billerica, MA) following the manufacturer’s instructions. For protein extraction for LC–MS assay development and testing, all isolates were grown on blood agar plates (Remel, Lenexa, KS) for 18–24 h at 35 °C with 5% CO_2_, and lysed with formic acid (FA) and acetonitrile (ACN) as described previously [21]. Briefly, for each sample, a 10 μL loop of fresh bacterial cells was resuspended in 0.5 mL 70% ethanol, vortexed for 1 min and centrifuged at 20,800×*g* for 2 min. Supernatant was removed and the pellet was resuspended in 100 μL of 70% FA and mixed to homogeneity, followed by addition of 100 μL of 100% ACN. The resulting solution was re-vortexed for 10 s and centrifuged for 2 min at 20,800×*g*. 150 μL of supernatant (FA/ACN lysate) was stored at − 20 °C for later use. For replicates in the test set, the preceding steps were performed three times from different regions of the same culture plate.

*Analysis of MCR*-*1 sequences* The protein sequences of 12 MCR-1 variants as shown in Table 2 were downloaded (http://www.ncbi.nlm.nih.gov/protein Accessed June, 2017).

**Table 2:** Protein sequences of MCR-1, MCR-2, MCR-3 and MCR-4 and their variants used for peptidomic analysis

MCR-1 and variants			Other MCR variants	
Protein name	NCBI accession*	Mutation	Protein	NCBI accession*
MCR-1	WP_049589868.1		MCR-2	WP_065419574.1
MCR-1 family	WP_072652801.1	H466N	MCR-2.1	WP_078254299.1
MCR-1 family	WP_076604686.1	W8C	MCR-3	WP_094313523.1
MCR-1 family	WP_065203556.1	N311K, L326S, I323F	MCR-3.9	AST36144.1
MCR-1.2	WP_065274078.1	Q3L	MCR-3.7	AST36141.1
MCR-1.3	WP_077064885.1	I38V	MCR-3.8	AST36143.1
MCR-1.4	WP_076611062.1	D440N	MCR-3.6	AST36140.1
MCR-1.5	WP_076611061.1	H452Y	MCR-3.5	ATP60693.1
MCR-1.6	WP_077248208.1	R536H	MCR-4	ASR73329.1
MCR-1.7	WP_085562392.1	A215T		
MCR-1.8	WP_085562407.1	Q3R		
MCR-1’	APY22148.1	missing 1M		

*NCBI accession and protein names in NCBI (Current as of 3/10/18)

Core (common) tryptic peptides were defined as those tryptic peptides present in all 12 MCR-1 sequences listed in Additional file 1: Table S1. Sequences were aligned with MultAlin (http://multalin.toulouse.inra.fr/multalin/multalin.html) and in silico tryptic digestion was performed using GPMAW10 (Lighthouse data, Denmark). A Microsoft Excel visual basic script was used to identify core tryptic peptides. Manual examination of the sequence alignment of the 12 MCR-1 variants and MCR-2, MCR-3, MCR-4 protein families was used to confirm the identification of unique tryptic peptides for MCR-1 variants. The uniqueness of the identified tryptic peptides to MCR-1 was analysed using both the Unipept Peptidome Analysis web tool (http://unipept.ugent.be/peptidefinder) and protein blast (https://blast.ncbi.nlm.nih.gov/Blast.cgi?PAGE=Proteins) during June 2017–December 2017.

*Tryptic protein digestion* A mixture of 8 μL of deionized H_2_O and 2 μL of FA/ACN lysate in a 1.6 mL microcentrifuge tube was frozen briefly on dry ice and lyophilized using a SpeedVac concentrator (Savant) with a refrigerated vapour trap (Savant RVT4104) and a vacuum pump (TRIVAC, Oerlikon Leybold Vaccum, Germany) for 20 min. The intact proteins were re-suspended in 96 μL of 100 mM NH_4_HCO_3_ and vortexed briefly. Then samples were sonicated (Qsonica Q500) for 2 min with 20 s on and 10 s off at 40% amplitude in an ice bath. Rapid trypsin digestions were carried out in a water bath for 15 min at 55 °C as described previously [22] with the addition of 4 μL of 0.1 μg/μL Trypsin or Trypsin/Lys-C as noted in the text (Promega, Madison, WI) in 100 mM NH_4_HCO_3_. Samples were spun briefly and then transferred to a 0.5 mL 0.22 μm Ultrafree centrifugal filter (Merck Millipore, MA) for 3 min filtration at 12,000×*g*. 10 μL of the pass-through fraction was used for total peptide concentration measurement using Qubit 2.0 Fluorometer (ThermoFisher, San Jose, CA). If the concentration was > 100 μg/mL, the digests were diluted to 100 μg/mL using 100 mM NH_4_HCO_3_ as the diluent.

*Labelled peptides* Peptides with > 95% purity containing heavy isotopic labels in R (U-13C6; U-15N4) or K (U-13C6; U-15N2) C-terminus amino acids were purchased from New England Peptide Group (Gardner, MA). Their characterization and concentration were provided by the manufacturer.

*Protein identification by Orbitrap LC*–*MS/MS* Bottom-up protein identification was carried out using an Orbitrap Lumos mass spectrometer (ThermoFisher Scientific) as previously described [22]. LC–MS/MS data were searched against a custom FASTA database composed of *E. coli* proteins (total of 4212 sequences downloaded from Uniprot.org in July 2016) and 9 MCR-1 sequences (MCR-1, and MCR-1.2–MCR-1.8) by Proteome Discoverer 1.4 (ThermoFisher) and Scaffold 4 (Proteome Software Inc., Portland, Oregon) as previously described [21, 22].

*ESPPredictor* The ESPredictor online web tool (http://software.broadinstitute.org/cancer/software/genepattern/esppredictor) was used as a guide to predict which tryptic peptides of MCR-1 would be most likely to be efficiently ionized and readily detected for MRM assay development [23].

*Targeted LC*–*MS/MS* Targeted LC–MS/MS was run on an Orbitrap Lumos mass spectrometer as described previously [22]. The acquisition time was set to 120 ms and gain was set to 2 × 10^5^. Skyline 3.7 software package (MacCross lab) was used for quantitative and relative spectral intensity comparisons.

*MRM assay* The MRM assay was run on an Agilent CubeChip 6495 QQQ with a high capacity 160 nl 150 mm chip (Agilent G4240-62010). The mobile phases were 0.1% FA, 5% ACN in H_2_O (A), and 0.1% FA, 5% H_2_O in ACN (B). The gradient was run from 5% to 20% B over 7 min with a flow rate of 0.4 μL/min. Dwell time was set to 20 ms for all transitions. The MS1 resolution and MS2 resolution were set to 0.7 Dalton. Other MS settings included gas temperature: 200 °C; Gas flow: 11 L/min; Delta EMV+: 300 V; flush volume: 8 μL and cell accelerator voltage: 2 V. Table 3 lists the peptides and transitions as well as collision energy for each transition. The three labelled peptide concentrations in the labelled peptide mix were 5 fmol/μL, 25 fmol/μL and 25 fmol/μL for DTFPQLAK, SVPAFFWTDK and ADHVSFNGYER respectively, based on the manufacturer’s determination of concentration. 2 μL of labelled peptide mix was added to 18 μL of digested peptide solution in a silanized vial (National C4000-S9, Thermo), and 4 μL was injected to the LC–MS. Between the sample runs, a no-matrix “blank” with 0.4 μL of labelled peptide mix was injected to the column as a quality control measure to monitor the LC–MS performance during batched runs. These no-matrix banks also served to wash the column and minimize carry-over effects of native positive peptides. As a further control measure, the column was washed and re-developed with a 30-min washing protocol after every 25-sample runs and 25-blank runs. The spectral library for MCR-1 peptides was created using the MS/MS spectra from Orbitrap Lumos.

**Table 3:** Tryptic peptides and transitions used in MRM assay

Peptide	Charge	Precursor (Da)	*T*1	*T*2	*T*3	*T*4	*T*5	*T*6
DTFPQLAK	2+	460.2478	y5 + (16.7)	y6 ++ (16.7)	y3 + (22.7)	y6 + (22.7)	y5 ++ (16.7)	
			556.3453 Da	352.2105 Da	331.2340 Da	703.4137 Da	278.6763 Da	
SVPAFFWTDK	2+	599.3006	y8 + (22)	y6 + (28)	y4 + (34)	y7 + (31)	y5 + (28)	y3 + (34)
			1011.4934 Da	843.4026 Da	549.2667 Da	914.4407 Da	696.3352 Da	363.1874 Da
ADHVSFNGYER	2+	647.7942	y7 + (29.2)	y8 + (29.2)	y9 + (29.2)	y6 + (29.2)		
			872.3897 Da	971.4581 Da	1108.5170 Da	785.3577 Da		

(): collision energy in parentheses

## Results

*Prediction of theoretical core peptides for MCR*-*1 and its variants* Table 2 lists MCR-1 and the 11 described sequence variants studied in this work. Using peptidomic analysis, 22 core tryptic peptides of MCR-1 and its variants were found (Additional file 1: Table S1). As the intention of this work was to design an MCR-1 specific assay, tryptic peptides shared by other MCR protein families were eliminated. Unique peptides were identified by protein blast and lowest common ancestor (LCA) analysis (https://unipept.ugent.be/datasets). Only those core peptides that were also unique to MCR-1 were considered for LC–MS/MS assay development.

**Fig. 1:**
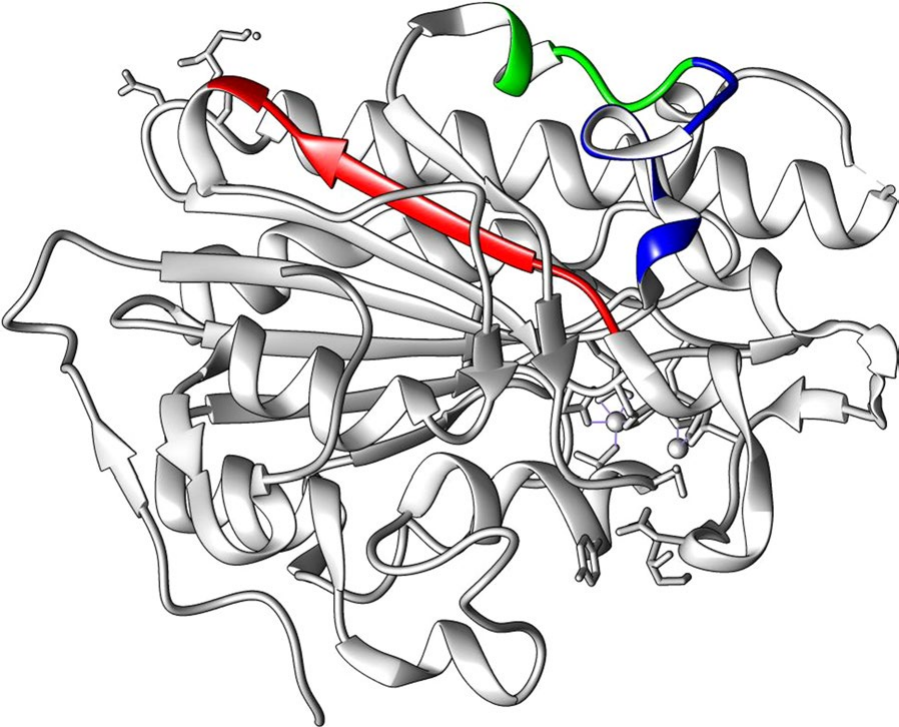
The locations of three chosen tryptic peptides in the MCR-1 crystal structure (PDB accession number 5GRR). DTFPQLAK is presented in green, SVPAFFWTDK in red and ADHVSFNGYER in blue. Figure made with UCSF Chimera

*Experimental detection of theoretically*-*determined tryptic peptide markers* Among the theoretically-determined core peptides unique to MCR-1 and its variants, we sought to identify peptides that were efficiently ionized and readily detectable by LC–MS/MS [23]. For experimental method development, we chose one *mcr*-*1*-containing *E. coli* isolate (P1, Table 4) that had been previously sequenced. A bottom-up proteomics (data-dependent acquisition (DDA)) analysis was performed using 1 μg protein that was digested with either trypsin alone or trypsin/Lys-C. This analysis found two high-quality MCR-1 peptides (DAVQATKPDMR, ADHVSFNGYER) with only single spectrum for each. Given the small number of peptides detected using the DDA approach, a targeted approach based on theoretical prediction made by ESPPredictor [23] was used. Using an ESPPredictor value threshold of > 0.3, five additional MCR-1 core peptides (VDYPTWGK, SYVNPIMPIYSVGK, DTFPQLAK, DVGMLVGLDDFVAANNGK and SVPAFFWTDK) that had not been detected by DDA were selected for targeted LC–MS using Orbitrap Lumos. Among these five peptides, only DTFPQLAK (ESP value 0.76) and SVPAFFWTDK (ESP value 0.42) were detected by targeted LC–MS. Thus, four peptides in total (DAVQATKPDMR, ADHVSFNGYER, DTFPQLAK, and SVPAFFWTDK) were initially selected as potential targets for assay development. However, DAVQATKPDMR was subsequently dropped from further study due to observed instability in the labelled DAVQATKPDMR peptide during MRM assay development. Figure 1 shows the locations of the three peptide markers chosen for final assay development in an MCR-1 crystal structure (PDB Accession 5GRR). MS/MS spectra as acquired by Obitrap Lumos LC–MS are provided for these three peptide markers in Additional file 1: Fig. S1 for reference.

**Table 4:** rdotp and *R* ratio values for isolates used in assay development and *mcr*-*1*-containing isolates in validation set

Assay	Group	Sample	rdotp value/*R* ratio value		
			DTFPQLAK	SVPAFFWTDK	ADHVSFNGYER
Method development	*mcr*-*1*	P1	0.98/1.17	0.99/0.20	1.0/0.45
		P2	0.99/0.95	0.98/0.19	1.0/0.34
		P3	0.99/1.27	0.99/0.26	1.0/0.47
		P4	0.99/1.13	0.99/0.22	1.0/0.38
		P5	0.96/0.27	0.96/0.07	0.98/0.13
	Negatives	N1	0.43/0.01	0.49/0.003	0.41/0.24
		N2	0.61/0.01	0.76/0.01	0.91/0.10
Method test	Blinded unknowns	U9	0.97/0.12	0.99/0.08	1.0/0.07* (0.9/0.09)
		U18	0.98/0.31	0.95/0.34* (0.76/0.5)	0.99/0.20
		U25	0.99/0.43	0.88/0.64* (0.71/0.9)	0.99/0.32
		U33	0.98/0.14	0.99/0.14	1.0/0.11* (0.75/0.18)
		U42	0.98/0.17	0.98/0.14	0.93/0.17* (0.74/0.27)
		U65	0.96/0.18	1.0/0.08	0.99/0.11* (0.91/0.14)
		U66	0.96/0.16	0.99/0.08	0.99/0.09* (0.79/0.15)
		U74	0.98/0.13	1.0/0.1	0.99/0.07* (0.76/0.12)
		U100	0.98/0.22	0.93/0.21* (0.74/0.32)	1.0/0.09

*After removal of one transition. The numbers in paranthese () are unadjusted values

*MRM assay development using Agilent ChipCube QQQ* We selected five *mcr*-*1*-containing isolates and two negative control isolates to develop the MRM assay (Table 4). Note that four of the *mcr-1*-containing isolates were collected from a single patient and were essentially clonal with the exception of different numbers of copies of the *ISApl1* insertion sequence [2, 20]. Labelled peptides were used to determine gradient setting, collision energy and amounts of labelled peptides added to digested samples.

We next assessed sample-to-sample carry-over, performance, and stability for the chosen peptide set. No detectable carry-over for any of the selected peptides was observed between samples during assay development. To monitor LC–MS performance and stability, a no-matrix blank containing the labelled peptide mix (at the same concentration as was used in the sample runs) was run interleaved between samples. These interleaved blanks served as both quality control and stability indicators for the instrument by monitoring the retention time variation and signal intensity variation. Additionally, these blanks served as HPLC column clean-up to minimize the chances of carry-over.

Among the five *mcr*-*1*-containing isolates used for test development (P1-P5), DTFPQLAK had the highest quality signal. The integrated peak areas observed in the 5 MCR-1-containing samples studied during test development ranged from 59 to 310 k for DTFPQLAK, 8–21 k for SVPAFFWTDK and 3–10 k for ADHVSFNGYER. Observed background noise for the two negative controls range was at or below the instrument detection limit for DTFPQLAK, 0.2–0.5 k for SVPAFFWTDK, and 3–7 k (higher values were due to nearby interfering peaks that were included in the integration window) for ADHVSFNGYER. Background signals in SVPAFFWTDK were due to interferences that were easily recognized by spectral inspection. On the basis of background noise considerations, DTFPQLAK was assessed as the best peptide marker for MCR-1.

**Fig. 2:**
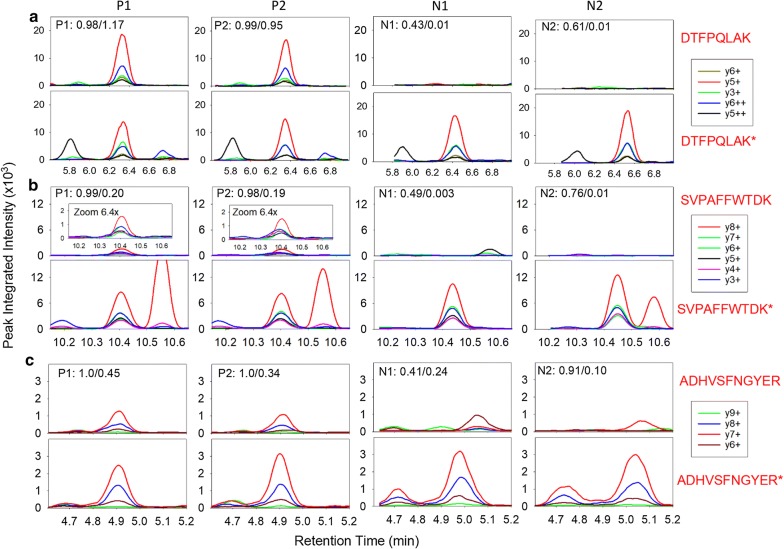
LC-MS chromatograms of **a** DTFPQLAK, **b** SVPAFFWTDK and **c** ADHVSFNGYER for two representative *mcr*-*1*-containing isolates (*P*1 and *P*2) and two negative controls (*N*1 and *N*2) used for method development. The rdotp/*R*-ratio for each peptide is shown in the respective box. Additional related data are contained in Additional file 1: Fig. S2a–c

Figure 2a shows the LC–MS chromatograms of DTFPQLAK for two *mcr*-*1*-containing isolates (P1 and P2) and two negative controls (N1 and N2) used for method development (Additional file 1: Fig. S2a). In the course of study of this peptide, it became apparent that the y3 + transition in the labelled peptide was higher in intensity than in the native peptide and may have been subject to interference, which occurs more frequently with shorter fragments. This effect was taken into account in manual expert review during the test phase below. In contrast, the intensity of the second peptide, SVPAFFWTDK, was an order of magnitude less than that of DTFPQLAK (Fig. 2b, Additional file 1: Fig. S2b), but still high enough for robust detection. In *mcr*-1-containing test isolate P5, this peptide demonstrated distortion in the y3 transition, likely due to interference, as well as a fully interfering transition (y6 in Additional file 1: Fig. S3).

The third peptide, ADHVSFNGYER demonstrated similar intensity to SVPAFFWTDK (Fig. 2c, Additional file 1: Fig. S2c). A small peak of variable magnitude with similar transitions at retention time of 4.7 min was observed nearby to both labelled and native ADHVSFNGYER peptide (whose retention time was 4.9 min). Interfering peaks were also observed for negative control isolate N1; these were distinguishable from true signals, as the transition rank order and retention time did not match to those from labelled peptide. The ratio dot product (rdotp), representing the normalized dot product of the light transition peak areas of the native peptide with the heavy transition peak areas of the labelled peptide, was only 0.41 for ADHVSFNGYER in isolate N1, indicating that the peaks observed were likely due to interference. For negative isolate N2, a single y7 peak in the sample was observed which matched to the y7 peak in the labelled peptide. However, the other two y6 + and y6 ++ peaks were not observed in N2. Consequently, the y7 peak in isolate N2 was judged to represent interference rather than signal from ADHVSFNGYER. Despite these observed interferences, we judged that the signals obtained indicated the chosen tryptic peptides were adequate for use in the assay.

Table 4 lists the rdotp and *R* ratio values obtained from Skyline [24] for the test development isolates. As noted above, rdotp scores quantify the spectral similarity between the native peptide and isotope labelled peptide. The R-value, in turn, quantifies the native peptide relative to the known concentration of isotope labelled peptide. Based on these data, we developed a set of expert rules for *mcr-1*-containing and negative calls in an attempt to build automaticity into the assay. It should be noted that the limited number of MCR-1 isolates in the development did not allow for rigorous model-fitting to develop optimized thresholds. The rules that follow were based on manual-fitting of rdotp and R thresholds to results from the development set spectra. The absolute amount of DTFPQLAK, SVPAFFWTDK, and ADHVSFNGYER labelled peptide added to each sample was 2 fmol, 10 fmol, and 10 fmol, respectively. DTFPQLAK was automatically called positive when rdotp ≥ 0.95 AND *R* ≥ 0.5. SVPAFFWTDK was automatically called positive when rdotp was ≥ 0.95 AND *R* ≥ 0.1. ADHVSFNGYER was automatically called positive when rdotp ≥ 0.95 AND *R* ≥ 0.2. All three peptides were automatically called negative when rdotp ≤ 0.85 AND *R* ≤ 0.05. Manual expert review was triggered for all signals falling between the automatic call criteria (for signals neither automatically positive nor automatically negative). During manual review, several factors were examined including MS/MS spectral transition rank order and retention time in comparison to the labelled peptide. Transitions attributable to interference were allowed to be manually removed, followed by recalculation of the rdotp and *R* ratio. However, in the end, the calls on the manually-reviewed spectra relied on the judgement of expert operators who were blinded to specimen identity. To quantify potential inter-operator variability and the effects of differences in judgment in the manual component of these calls, manual review was performed independently by three blinded operators. For an isolate to be declared MCR-1 positive, at least two of three peptide markers had to be called positive independently, either by automatic rules or by manual review.

*Blinded method test set* To evaluate the feasibility of our assay, we constructed a blinded test set of 99 de-identified clinical isolates consisting of three additional *mcr*-*1*-containing isolates not used in the assay-development set that were tested in triplicate (9 samples) and 90 negative control isolates (Table 1). The *mcr-1*-containing isolates were randomly distributed and all 99 runs were treated independently. Collection of LC–MS/MS data was performed by a single expert operator who was blinded to the identity of the samples. As explained above, analysis of data requiring manual review by expert rule criteria was performed by three independent blinded operators. The list of determinations for 99 measurements from each of the three operators was compared to a result key by a fourth independent operator, who also prepared the key.

The test set was run in two batches. A single negative sample used in the test development phase was included in the first batch. The operators were blinded to which sample this was, and it was correctly called negative and not included in the sensitivity and specificity calculations. Additional file 1: Fig. S4 shows the intensities and retention times for the three labelled peptides in the first 49 samples and 50 no-matrix blank runs.

Analysis demonstrated that the three labelled peptides in the first 49 samples had adequately stable apex retention times of 6.4 ± 0.1 min for DTFPQLAK, 10.43 ± 0.03 min for SVPAFFWTDK, and 5.07 ± 0.05 min for ADHVSFNGYER. The retention times of the three peptides for the first batch of 50 no-matrix blanks shifted by a statistically significant, but analytically negligible, amount to 6.79 ± 0.05 min for DTFPQLAK, 10.51 ± 0.02 min for SVPAFFWTDK, and 5.32 ± 0.04 min for ADHVSFNGYER. The signal intensities for labelled peptides in the first 49 samples varied and were generally lower than that in blank runs, which we attributed to matrix effects. The average signal intensity for DTFPQLAK was 215 K in samples and 429 K in blanks. For ADHVSFNGYER, it was 15 K in samples and 20 K in blanks. For SVPAFFWTDK, it was 38 k in samples and 221 k in blanks, the most significant reduction due to matrix affects in the set (Additional file 1: Fig. S4).

*Test set performance* Expert rules were applied to the 3 peptides in each of 99 samples (297 peptides total). 122/297 peptides were correctly called negative by the automatic call rule. 0/297 peptides were called positive by the automatic rule. The remainder of the peptides (175) fell between the automatic positive and negative call rules and qualified for expert manual review. For manual classification, overall assignment of a sample as MCR-1 positive or negative was made once the minimum of two peptides were individually classified as positive or negative. In cases where one of the three peptide calls was discordant, the final call was made on the basis of the two concordant individual peptide calls, as indicated above. Sensitivity and specificity are given for the overall calls, not individual peptide calls. Manual review by three independent blinded expert operators correctly identified all *mcr*-*1*-containing samples (9/9 correct identifications), on the basis of retention time and transition rank order criteria. No false positive calls were made for the 90 negative controls by any of the operators, yielding an overall performance of 100% sensitivity and 100% specificity for detection of MCR-1 protein in the blinded test set. Table 4 shows the rdotp values and *R* ratios for the 9 isolates that were identified as MCR-1 positive. Compared with the values obtained from assay development isolates, the *R* ratios for these 9 *mcr*-*1*-positive samples were threefold lower, possibly attributable to lower expression.

*Post*-*analysis examination of expert rules* After the analysis of all 99 samples, we re-examined the expert rules using the single best peptide marker DTFPQLAK to determine if a higher percentage of automatic correct positive calls could have been obtained with different thresholds. We found that if the rule for positive calls had been set at: rdotp > 0.95 AND *R* > 0.12, this would have resulted in all 9 positive samples called correctly, with no false positive calls. Rigorous testing of this rule would require re-evaluation with an independent second test set, which was not performed here.

## Discussion

In this work, we sought to demonstrate the feasibility of a mass spectrometry-based method for the rapid detection of the MCR-1 protein directly in cultured clinical isolates. Using a genoproteomic approach that combines theoretical peptidome analysis with experimental LC–MS/MS [19, 22], we selected three efficiently ionized and detected tryptic peptides specific to the MCR-1 protein that could be detected by LC–MS/MS following rapid tryptic digestion of *mcr*-*1*-containing isolates. The three peptides chosen for this assay are not shared by other MCR protein families identified at the time of this writing (Additional file 1: Table S1).

To characterize the feasibility and performance of this method, we constructed a test set containing three additional *mcr*-*1*-containing isolates not used in the assay development set and prepared in triplicate and 90 negative control clinical isolates. A combination of rule-based calls and manual evaluation of intermediate values by three independent, blinded operators identified all *mcr*-*1*-containing isolates with 100% sensitivity and 100% specificity (9/9 positive identifications, 90/90 negative identifications). Though the number of publicly available *mcr*-*1*-containing isolates in the United States (where this work was performed) is currently limited, this proof-of-concept study demonstrates feasibility and provides the basis for application in a larger set of *mcr*-*1*-containing isolates.

Our method differs from other commonly used tryptic digestion methods in a few respects that are important to point out. First, our assay employs a rapid tryptic protein digestion technique without protein denaturation, reduction/alkylation, overnight digestion or desalting, steps that would be difficult to implement as part of a routine workflow in a clinical microbiology laboratory. The general details of this protocol have been reported previously, but with a few important modifications described as follows. The lysate volume was reduced to 2 μL, producing a yield of 5–20 μg digested peptide product (data not shown). Lyophilization and sonication steps were added to improve protein resuspension after removal of FA and ACN. To achieve this, lyophilization was performed for 20 min and we believe may be superior to simple drying with a SpeedVac concentration. Successful lyophilization was confirmed by the presence of a visible white precipitate on the bottom of the tube. A rapid Qubit protein concentration measurement was performed to allow more precise control of the peptide concentration that was loaded into the column, and a 0.22 μm filtration step was included to prevent undissolved peptides or particles in the sample from clogging the microfluidic system in the ChipCube.

One of our findings was that the MCR-1 protein was present at very low concentrations in the protein extracts from all isolates requiring a highly sensitive LC–MS instrument for its detection. We sought to develop a set of criteria based on rdotp and R values to allow fully automatic identification of positive samples during the assay development phase; however, the MCR-1 protein was found to be present in at least threefold lower concentration in the test set than in the assay development isolates. This resulted in lower R values but with rdotp values still meeting criteria established for automatic positive and negative calls in most cases. However, as the automatic call criteria required that both the R-value and the rdotp values met threshold values, all of the *mcr*-*1*-containing samples in the test set fell into the intermediate range that was reflexed to expert manual review. Future improvements in the assay by enrichment procedures may improve the signal-to-noise ratio and enhance its sensitivity, potentially allowing for expert call rules that discriminate between positive and negative samples without manual review [25, 26]. As we noted, retrospective analysis demonstrated that had the rule rdotp > 0.95 AND *R* > 0.12 been employed for positive calls, all true positives would have been correctly called with no incurred false positive calls. However, this rule would require testing on an independent test set, not performed here.

The ADHVSFNGYER peptide contains an asparagine (*N*) amino acid in the sequence which can undergo spontaneous deamidation. Using QTOF analysis (data not shown), we observed deamidated product in the labelled peptide, with deamidated ADHVSFNGYER* comprising 25% of the total ADHVSFNGYER* spectra. An accompanying small peak (approximately 28%) with the same *m/z* of ADHVSFNGYER* was also observed. The exact sequence corresponding to this small peak was unknown, and it was considered as a possible contaminant. Deamidation of ADHVSFNGYER may affect its absolute quantification, but it should not affect the qualitative detection. Four transitions were initially selected for ADHVSFNGYER during MRM assay development (Table 3). After unblinding of the 99-sample test set results and further analysis, we found the y6 + transition had interference as shown in Additional file 1: Fig. S5a. Removal of y6 + improves the rdotp values, which would make the positive identification easier as shown in Additional file 1: Fig. S5b.

## Conclusions

In conclusion, we have demonstrated the feasibility of rapid detection of peptide markers of the MCR-1 protein in clinical isolates using LC–MS/MS, without lengthy sample processing. Total assay time from isolate to result is < 90 min. The best peptide marker evaluated in this work was DTFPQLAK. While the number of MCR-1 isolates included in the test set was necessarily limited due the small number of isolates currently available publicly in the United States, this study provides a proof-of-concept approach to the rapid detection of MCR-1, and forms the basis for development of rapid mass spectrometry methods for this important emerging resistance element.

## Additional file

**Additional file 1: Table S1.** Core peptides for 12 MCR-1 variants and ESP values per ESPPredictor. **Figure S1.** MS/MS spectra acquired by Orbitrap Lumos LC–MS for three peptide markers. **Figure S2a.** LC–MS chromatograms of DTFPQLAK for the five *mcr-1*-containing isolates (P1–P5) and two negative controls (N1 and N2) used in assay development. **Figure S2b.** LC–MS chromatograms of SVPAFFWTDK for the five *mcr-1*-containing isolates (P1–P5) and two negative controls (N1 and N2) used in assay development. **Figure S2c.** LC–MS chromatograms of ADHVSFNGYER for the five *mcr-1*-containing isolates (P1–P5) and two negative controls (N1 and N2) used in assay development. **Figure S3.** For *mcr-1*-containing isolate P5 used in assay development, distortion in the y3 transition, likely due to interference, as well as a fully interfering transition (y6) were observed. Left: original LC–MS chromatogram; Right: LC–MS chromatogram after removal of the interfering transition y6. **Figure S4.** Left panel shows peak intensity variation during the first 49-sample and 50 no-matrix blank runs. Right panel shows retention time variation during the first 49-sample and 50 no-matrix blank runs. The no-matrix blank run contained labeled peptides only were run between the samples and were used to monitor instrument performance. Strong matrix effect was observed for SVPAFFWTDK*. **Figure S5.** y6 interferences were observed in native ADHVSFNGYER peptide. Removal of the y6 transition increased the rdotp value for *mcr-1*-containing isolates. Ratios are given as rdopt/R ratio.
