# Supplementary material for: Rapid detection of colistin resistance protein MCR-1 by LC–MS/MS
Source: Clin Proteomics. 2019 Feb 26;16:8. doi: 10.1186/s12014-019-9228-2 (PMC6390366; doi:10.1186/s12014-019-9228-2)
Supplement: Supplementary file 1 — Additional file 1: Table S1. Core peptides for 12 MCR-1 variants and ESP values per ESPPredictor. Figure S1. MS/MS spectra acquired by Orbitrap Lumos LC–MS for three peptide markers. Figure S2a. LC–MS chromatograms of DTFPQLAK for the five mcr-1-containing isolates (P1–P5) and two negative controls (N1 and N2) used in assay development. Figure S2b. LC–MS chromatograms of SVPAFFWTDK for the five mcr-1-containing isolates (P1–P5) and two negative controls (N1 and N2) used in assay development. Figure S2c. LC–MS chromatograms of ADHVSFNGYER for the five mcr-1-containing isolates (P1–P5) and two negative controls (N1 and N2) used in assay development. Figure S3. For mcr-1-containing isolate P5 used in assay development, distortion in the y3 transition, likely due to interference, as well as a fully interfering transition (y6) were observed. Left: original LC–MS chromatogram; Right: LC–MS chromatogram after removal of the interfering transition y6. Figure S4. Left panel shows peak intensity variation during the first 49-sample and 50 no-matrix blank runs. Right panel shows retention time variation during the first 49-sample and 50 no-matrix blank runs. The no-matrix blank run contained labeled peptides only were run between the samples and were used to monitor instrument performance. Strong matrix effect was observed for SVPAFFWTDK*. Figure S5. y6 interferences were observed in native ADHVSFNGYER peptide. Removal of the y6 transition increased the rdotp value for mcr-1-containing isolates. Ratios are given as rdopt/R ratio. [file 12014_2019_9228_MOESM1_ESM.pdf]

SUPPLEMENTARY MATERIAL

Rapid Detection of Colistin Resistance Protein MCR-1 by LC-MS/MS

Honghui Wang, Yong Chen, Jeffrey R. Strich, Steven K. Drake, Jung-Ho Youn, Avi Z. Rosenberg, Marjan Gucek,

Patrick T. McGann, Anthony F. Suffredini, John P. Dekker

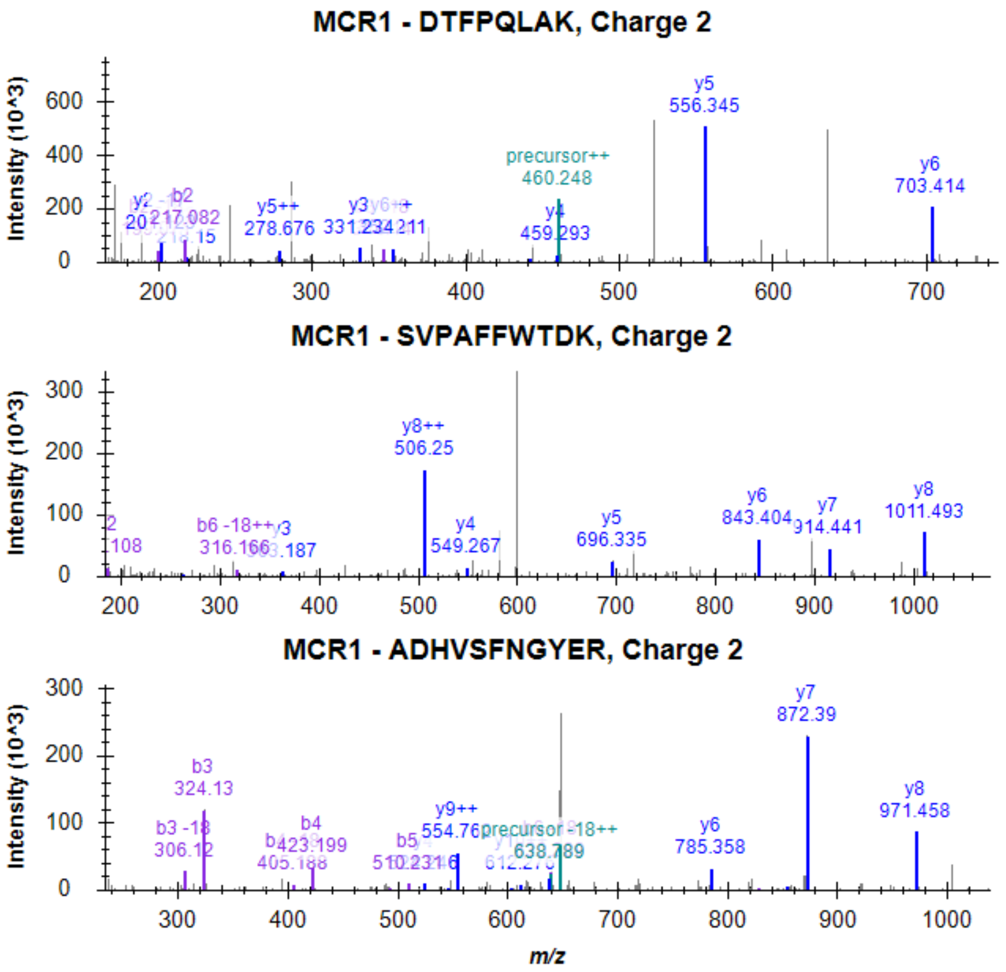

Supplemental Figure 1: MS/MS spectra acquired by Orbitrap Lumos LC-MS for three peptide markers.

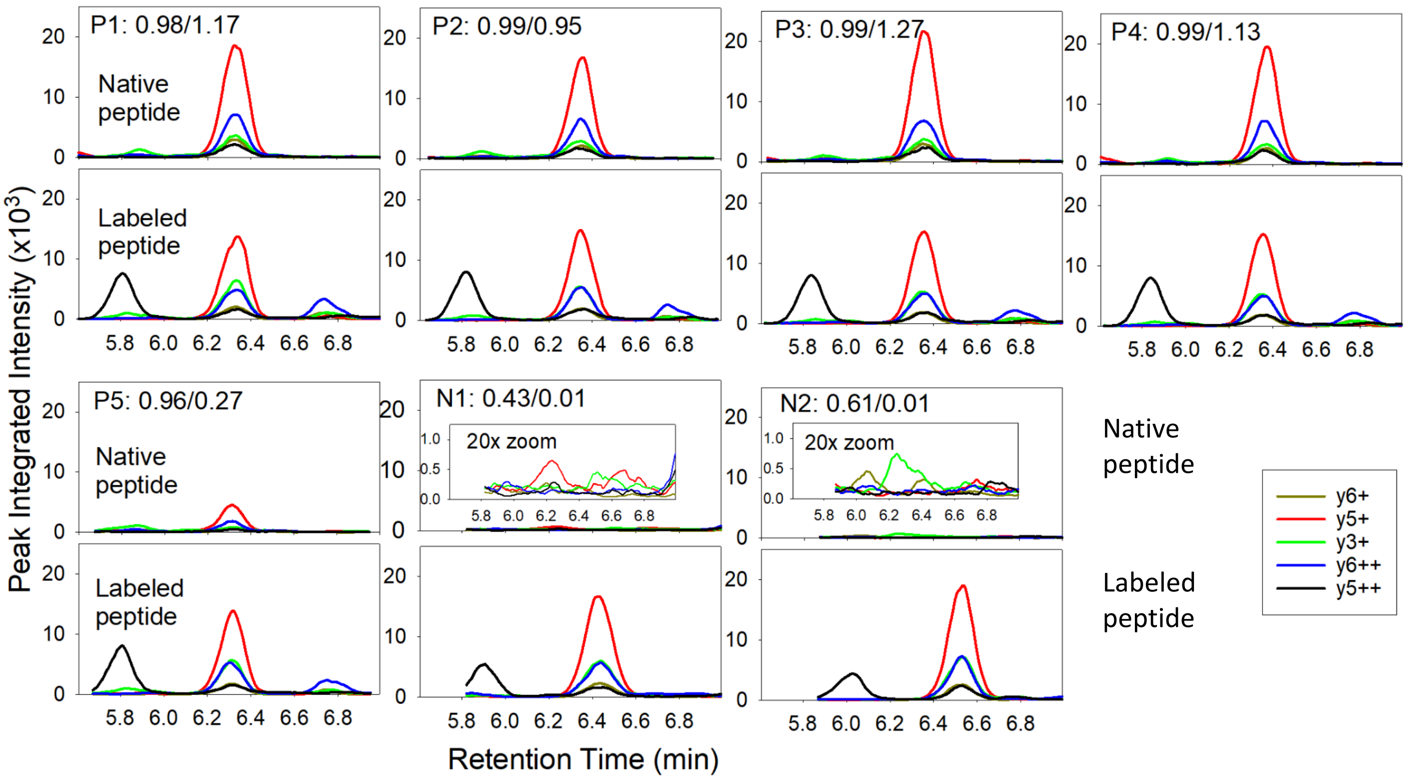

Supplemental Figure 2a: LC-MS chromatograms of DTFPQLAK for the five *mcr-1*-containing isolates (P1-P5) and two negative controls (N1 and N2) used in assay development.

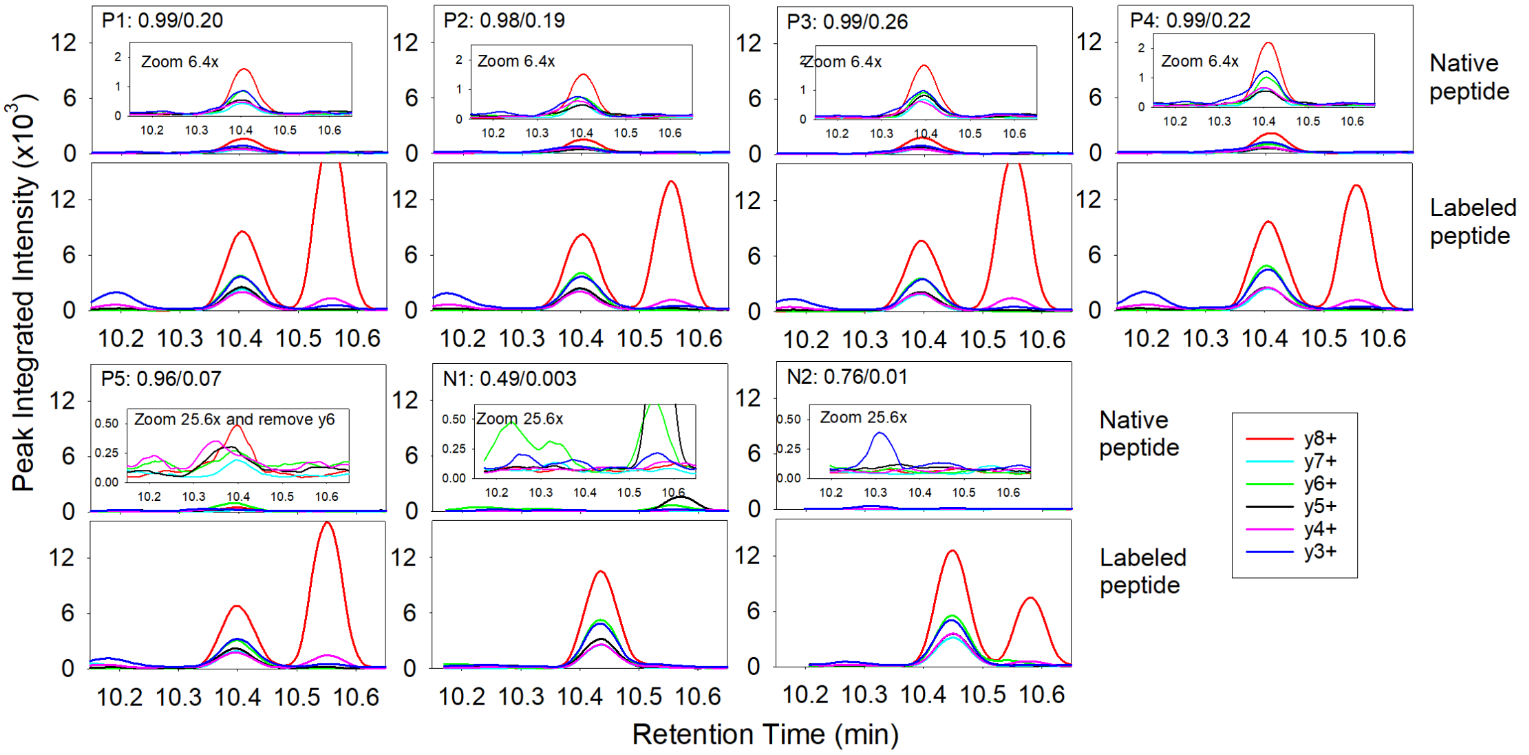

Supplemental Figure 2b: LC-MS chromatograms of SVPAFFWTDK for the five *mcr-1*-containing isolates (P1-P5) and two negative controls (N1 and N2) used in assay development.

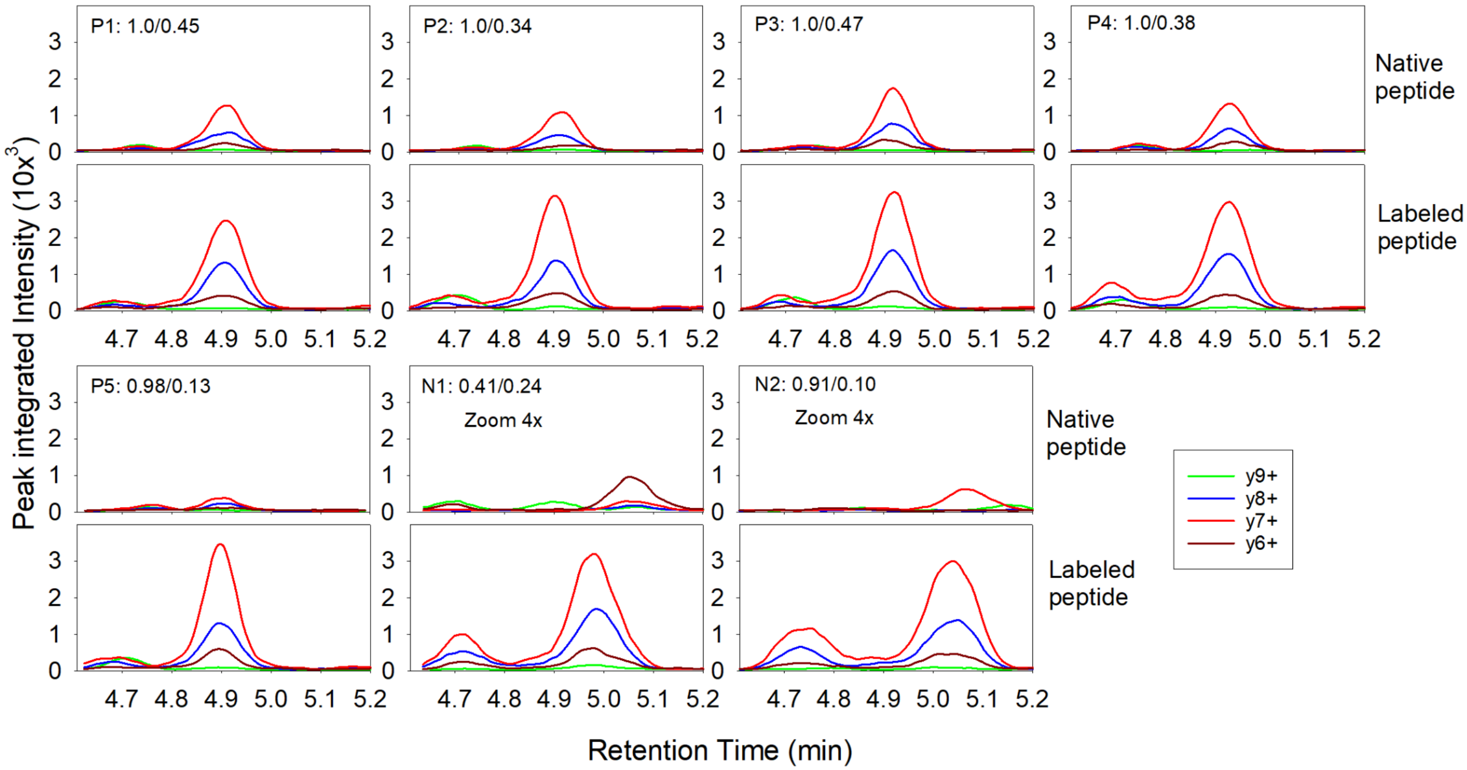

Supplemental Figure 2c: LC-MS chromatograms of ADHVSFNGYER for the five *mcr-1*-containing isolates (P1-P5) and two negative controls (N1 and N2) used in assay development.

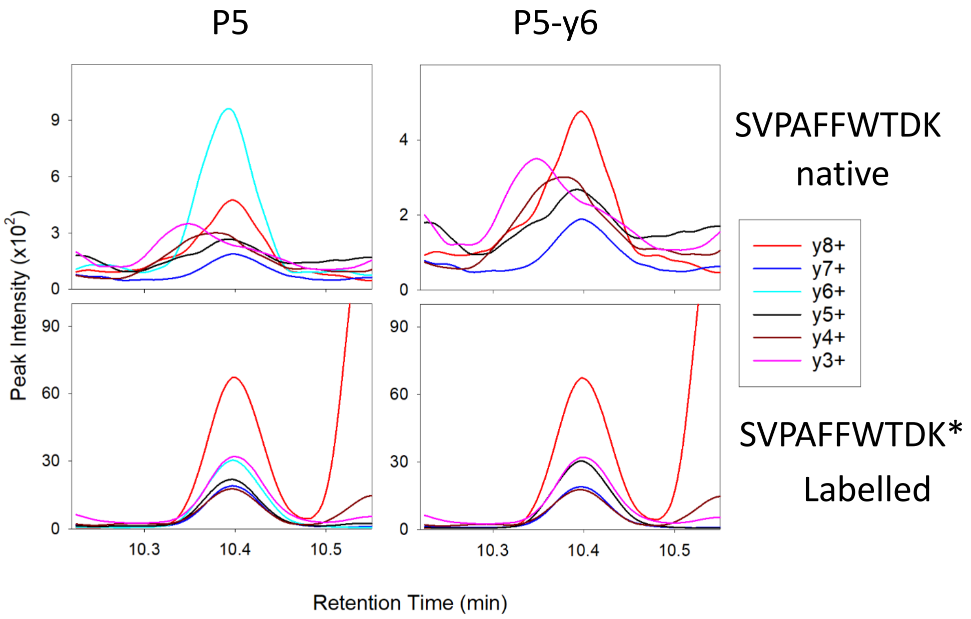

Supplemental Figure 3: For *mcr-1*-containing isolate P5 used in assay development, distortion in the y3 transition, likely due to interference, as well as a fully interfering transition (y6) were observed. Left: original LC-MS chromatogram; Right: LC-MS chromatogram after removal of the interfering transition y6.

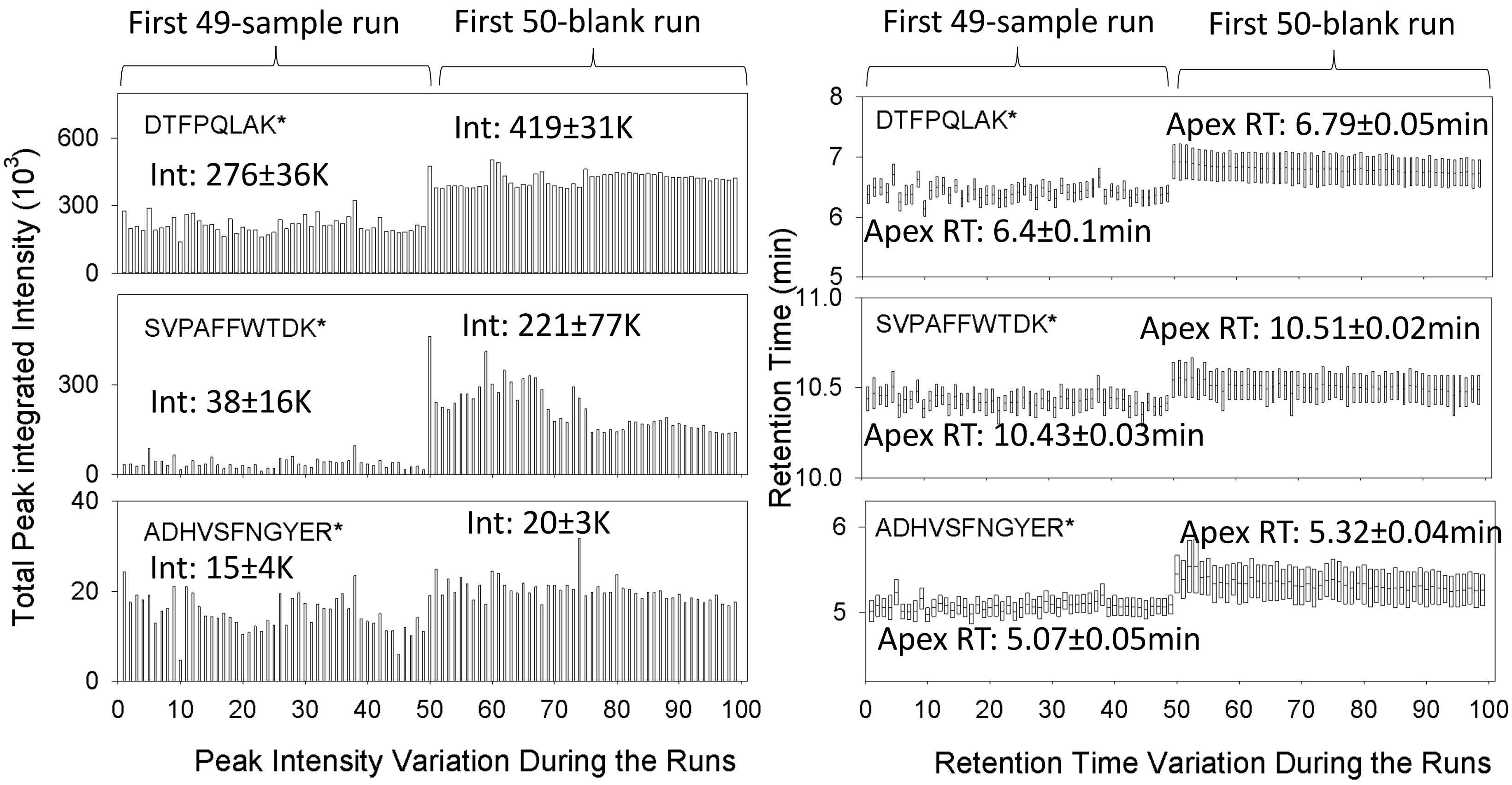

Supplemental Figure 4: Left panel shows peak intensity variation during the first 49-sample and 50 no-matrix blank runs. Right panel shows retention time variation during the first 49-sample and 50 no-matrix blank runs. The no-matrix blank run contained labelled peptides only were run between the samples and were used to monitor instrument performance. Strong matrix effect was observed for SVPAFFWTDK*

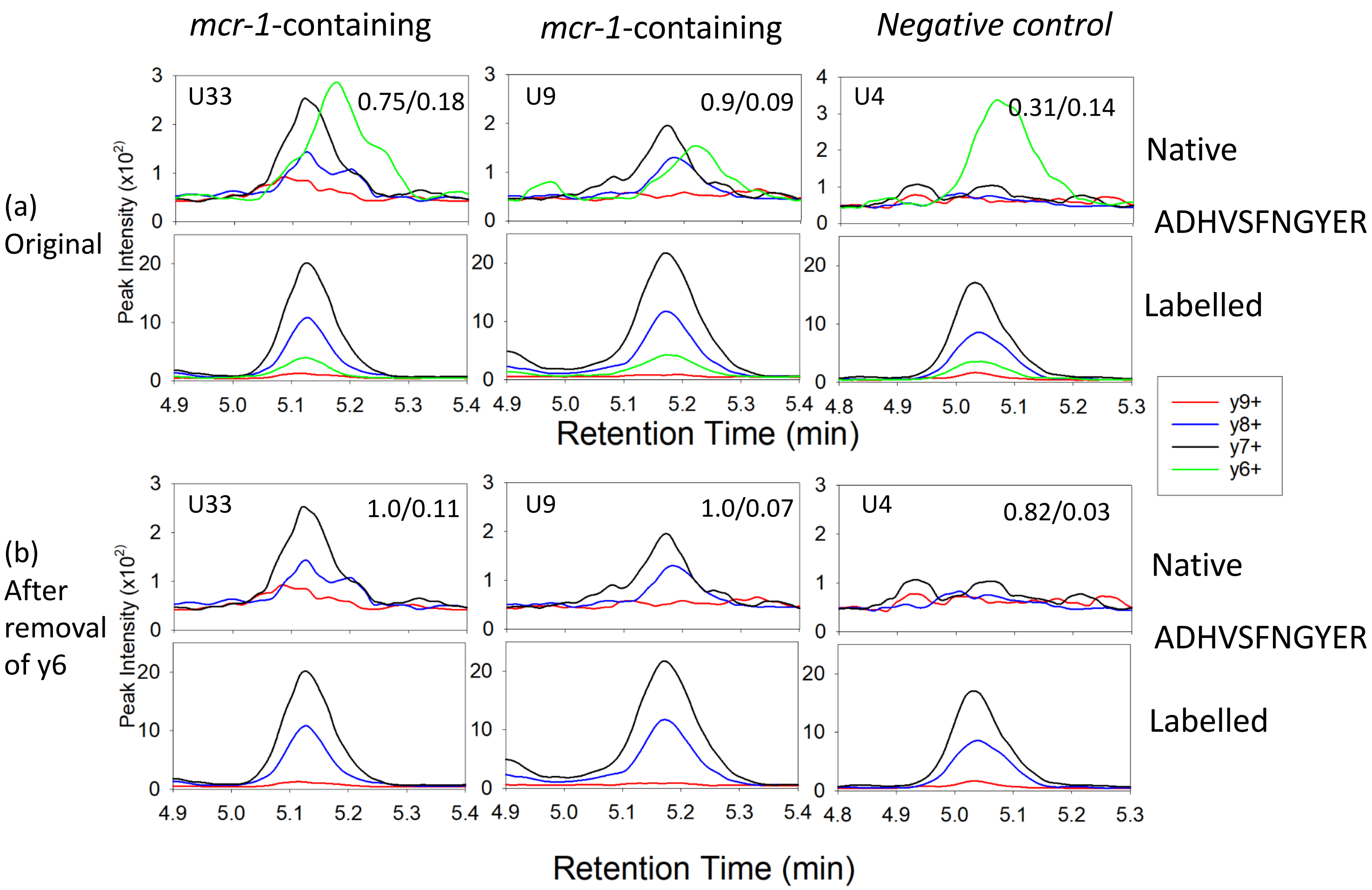

Supplemental Figure 5: y6 interferences were observed in native ADHVSFNGYER peptide. Removal of the y6 transition increased the rdotp value for *mcr-1*-containing isolates. Ratios are given as rdopt/R ratio.

**Supplemental Table 1:** Core peptides for 12 MCR-1 variants and calcuated ESPPredictor values (ESP)					
**In silico digested tryptic peptide**	**# variants containing peptide**	**aa position**	**Unique to MCR-1**	**Note**	**ESP**
DAVQATKPDMR	12	225-235	yes	Detected (not used)	0.40
ADHVSFNGYER	12	250-260	yes	Detected	0.28
DTFPQLAK	12	261-268	yes	Detected	0.76
SVPAFFWTDK	12	491-500	yes	Detected	0.42
SYVNPIMPIYSVGK	12		yes	Not detected	0.53
VDYPTWGK	12		yes	Not detected	0.32
DVGMLVGLDDFVAANNGK	12		yes	Not detected	0.30
SATNNAICNTNPYNECR	12		yes	Not detected	0.29
IIGLGVLPSLLVAFVK	12		yes	Not detected	0.24
DLLNAAFIMR	12		yes	Not detected	0.23
QTGITPMATDTVLTHDAITPTLLK	12		yes	Not detected	0.15
LGLIVASLALILLPVVAFSSHYASFFR	12		yes	Not detected	0.08
SVSPFVLVASVAVFLTATANLTFFDK	12		yes	Not detected	0.06
LFDVTADK	12		no	MCR-2.1	0.59
AQFADYK	12		no		0.19
DMLIMLHQMGNHGPAYFK	12		no	MCR-2	0.09
DNNSDSK	12		no		0.05
DTIYHAK	12		no		0.07
FTPVCEGNELAK	12		no	MCR-2	0.87
LASIEYK	12		no		0.28
LVVFVVGETAR	12		no		0.32
VHKPLR	12		no		0.11
LGVSILWR	11		yes		0.27
YQENVLDTLDR	11		no	MCR-2	0.61
MMQHTSVWYR	8		yes		0.12
DHTAFIR	1		no		0.22
HTSVWYR	1		yes		0.11
LGVSFLWR	1		yes		0.24
MMLHTSVWYR	1		yes		0.14
MMQHTSVCYR	1		yes		0.18
MQHTSVWYR	1		yes		0.10
VLDTSDR	1		no		0.09
